# Pro‐inflammatory dopamine‐2 receptor‐specific T cells in paediatric movement and psychiatric disorders

**DOI:** 10.1002/cti2.1229

**Published:** 2020-12-17

**Authors:** Deepti Pilli, Alicia Zou, Ruebena Dawes, Joseph A Lopez, Fiona Tea, Ganesha Liyanage, Fiona XZ Lee, Vera Merheb, Samuel D Houston, Aleha Pillay, Hannah F Jones, Sudarshini Ramanathan, Shekeeb Mohammad, Anthony D Kelleher, Stephen I Alexander, Russell C Dale, Fabienne Brilot

**Affiliations:** ^1^ Brain Autoimmunity Group Kids Neuroscience Centre Kids Research at the Children's Hospital at Westmead Sydney NSW Australia; ^2^ Discipline of Child and Adolescent Health Faculty of Medicine and Health The University of Sydney Sydney NSW Australia; ^3^ Genomic Medicine Group Kids Neuroscience Centre Kids Research at the Children's Hospital at Westmead Sydney NSW Australia; ^4^ School of Medical Sciences Discipline of Applied Medical Science Faculty of Medicine and Health The University of Sydney Sydney NSW Australia; ^5^ School of Biomedical Engineering The University of Sydney Sydney NSW Australia; ^6^ The Kirby Institute University of New South Wales Sydney NSW Australia; ^7^ Centre for Kidney Research Children's Hospital at Westmead Sydney NSW Australia; ^8^ Brain and Mind Centre The University of Sydney Sydney NSW Australia

**Keywords:** autoimmunity, autoimmune encephalitis, dopamine‐2 receptor antibodies, neurodevelopmental disorders, pro‐inflammatory T cells

## Abstract

**Objectives:**

A dysregulated inflammatory response against the dopamine‐2 receptor (D2R) has been implicated in movement and psychiatric disorders. D2R antibodies were previously reported in a subset of these patients; however, the role of T cells in these disorders remains unknown. Our objective was to identify and characterise pro‐inflammatory D2R‐specific T cells in movement and psychiatric disorders.

**Methods:**

Blood from paediatric patients with movement and psychiatric disorders of suspected autoimmune and neurodevelopmental aetiology (*n* = 24) and controls (*n* = 16) was cultured *in vitro* with a human D2R peptide library, and D2R‐specific T cells were identified by flow cytometric quantification of CD4^+^CD25^+^CD134^+^ T cells. Cytokine secretion was analysed using a cytometric bead array and ELISA. HLA genotypes were examined in D2R‐specific T‐cell‐positive patients. D2R antibody seropositivity was determined using a flow cytometry live cell‐based assay.

**Results:**

Three immunodominant regions of D2R, amino acid (aa)121–131, aa171–181 and aa396–416, specifically activated CD4^+^ T cells in 8/24 patients. Peptides corresponding to these regions were predicted to bind with high affinity to the HLA of the eight positive patients and had also elicited the secretion of pro‐inflammatory cytokines IL‐2, IFN‐ γ, TNF, IL‐6, IL‐17A and IL‐17F. All eight patients were seronegative for D2R antibodies.

**Conclusion:**

Autoreactive D2R‐specific T cells and a pro‐inflammatory Th1 and Th17 cytokine profile characterise a subset of paediatric patients with movement and psychiatric disorders, further underpinning the theory of immune dysregulation in these disorders. These findings offer new perspectives into the neuroinflammatory mechanisms of movement and psychiatric disorders and can influence patient diagnosis and treatment.

## Graphical abstract

In this study, we report of autoreactive dopamine‐2 receptor (D2R)‐specific T cells in a subset of paediatric movement and psychiatric disorders. These T cells recognised three immunodominant regions of D2R that were predicted to bind with high affinity to HLA class II molecules of patients, and T‐cell activation was associated with the secretion of pro‐inflammatory cytokines. These findings suggest a role for autoreactive T cells in the immune dysregulation of movement and psychiatric disorders.

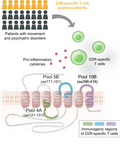

## Introduction

Recent years have seen a surge in evidence supporting autoimmunity as a cause of a subset of movement and psychiatric disorders.^1^, ^2^, ^3^, ^4^, ^5^, ^6^, ^7^ This paradigm has been largely driven by the discovery of antibodies against various neuronal proteins that have guided differential diagnosis and treatment regimens and led to an improved prognosis. One such neuronal protein targeted by antibodies is the dopamine‐2 receptor (D2R). In the brain, D2R is expressed abundantly in the basal ganglia, limbic system and cerebral cortex.^8^ Dopamine signalling in these neural networks primarily regulates voluntary action, motor control, learning and behavioural responses.^8^, ^9^ In the periphery, many immune cells also express dopamine receptors and may have immunomodulatory effects.^10^, ^11^ Given these roles, D2R has been implicated in movement and psychiatric disorders that have been associated with a dysregulated immune system.

Several studies have reported D2R antibodies in a subset of paediatric patients with movement and psychiatric disorders. These include disorders with a well‐recognised autoimmune aetiology, such as Sydenham's chorea and basal ganglia encephalitis, and also neuropsychiatric and neurodevelopmental disorders, such as first episode of psychosis, Tourette's syndrome and paediatric acute‐onset neuropsychiatric syndrome (PANS).^12^, ^13^, ^14^, ^15^, ^16^, ^17^, ^18^, ^19^ The precise mechanisms underlying autoimmunity in these disorders remain unclear, but it has been postulated that D2R antibodies may alter dopaminergic signalling by stimulating excess dopamine secretion,^20^ inducing inhibitory D2R signalling^16^ and triggering receptor internalisation.^19^ Additionally, D2R antibodies can be useful in identifying patients who can be amenable to immunotherapy.^17^, ^21^, ^22^, ^23^, ^24^, ^25^

The field has focused predominantly on aberrant antibody responses and less is known about the autoreactive T‐cell response against D2R in movement and psychiatric disorders. Cellular immunity is a major compartment of the adaptive immune response, and in particular, CD4^+^ T helper cells are key in orchestrating the effects of other immune cells and molecules. The central nervous system (CNS) is an accessible site for T cells. In the steady state, they traverse the CNS and actively partake in immunosurveillance. In the case of a murine model of post‐infection associated autoimmunity, reactive T cells migrate from the periphery into the CNS and contribute to neuroinflammation.^26^, ^27^, ^28^ Indeed, a dysregulated T‐cell compartment has been described in other neuroinflammatory disorders, most notably in multiple sclerosis (MS),^29^, ^30^, ^31^ but also in neuromyelitis optica spectrum disorders (NMOSDs),^32^, ^33^, ^34^ Rasmussen's encephalitis^35^, ^36^ and paraneoplastic syndromes.^37^, ^38^

In this study, we report on the presence of peripheral autoreactive T cells against D2R in paediatric patients with movement and psychiatric disorders. We identified three immunodominant regions of D2R, which induced T‐cell activation, were high‐affinity binders to HLA class II molecules and elicited pro‐inflammatory cytokine secretion. Notably, the presence of D2R‐specific T cells was not associated with D2R antibodies. Collectively, our findings inform the potential role of autoreactive T cells in movement and psychiatric disorders and provide new insights into the contribution of the immune system in neurodevelopmental disorders.

## Results

### T cells in patients with movement and psychiatric disorders recognised three immunodominant regions of D2R

**Figure 1:**
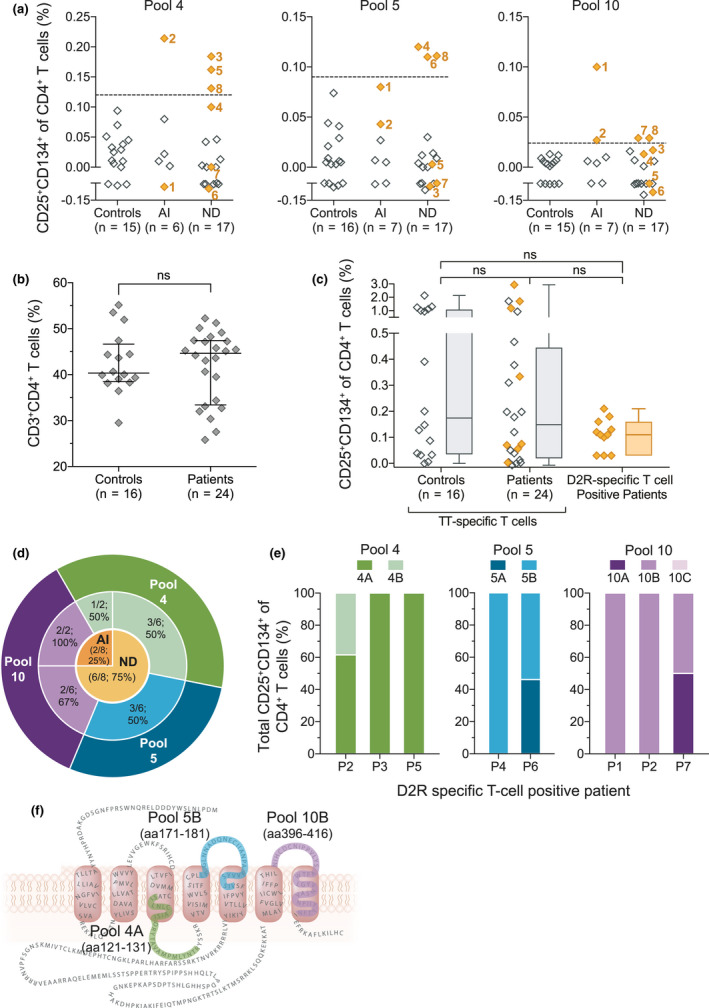
T cells in patients with movement and psychiatric disorders recognised dopamine‐2 receptor (D2R) at three distinct regions. D2R peptide pools that elicited T‐cell activation in patients with movement and psychiatric disorders of an autoimmune (AI) and neurodevelopmental (ND) aetiology were determined by assessing the co‐expression of CD25 and CD134 on CD4^+^ T cells using the CD25/CD134 assay. **(a)** Patient whole blood (*n* = 24) was stimulated with the 10 master pools of D2R peptides and eight patients (numbered orange diamonds) exhibited activation against at least one of three pools: pool 4, pool 5 and pool 10. Positive patients were defined as a frequency of CD25^+^CD134^+^CD4^+^ T cells above the threshold (dashed line; mean + 3SD of controls (*n* = 16)) for each peptide pool. Samples were tested once soon after their collection to preserve sample integrity. **(b)** The percentage of total CD3^+^CD4^+^ T cells in controls and patients was not significantly (ns) different (Mann–Whitney *U*‐tests, *P* = 0.86; error bars = mean ± SD). **(c)** Activation of antigen‐specific T cells in response to the recall antigen tetanus toxoid (TT) was tested concurrently to the activation of D2R‐specific T cells. The frequency of D2R‐specific T cells in patients was comparable to the frequency of TT‐specific T cells in controls, in patients with D2R‐specific T cells (orange diamonds) and in patients without D2R‐specific T cells (Kruskal–Wallis tests, *P* = 0.46; whiskers = minimum to maximum). **(d)** 6/8 D2R‐specific T‐cell‐positive patients had an ND aetiology, and 2/8 D2R‐specific T‐cell‐positive patients had an AI aetiology. **(e)** The peripheral blood mononuclear cells of 7/8 positive patients were re‐assessed against sub‐pool peptide of pools 4, 5 and 10. Due to sample limitation, samples were tested once, while ensuring all relevant sub‐pools were tested. Of the total activated D2R‐specific T cells, there was preferential activation by pool 4A (3/3), pool 5B (2/2) and pool 10B (2/3). **(f)** These three immunogenic sub‐pools corresponded to three discrete regions of thehuman D2R (green = pool 4A; blue = pool 5B; purple = pool 10B).

D2R‐specific T cells in paediatric patients with movement and psychiatric disorders (*n* = 24) were identified by determining CD4^+^ T‐cell activation when whole blood was stimulated with 10 master pools of human D2R peptides. The co‐expression of CD25 and CD134 on CD4^+^ T cells was assessed as a proxy for T‐cell activation.^39^ Compared to the control cohort, a 6.9‐ to 21.5‐fold higher frequency of activated D2R‐specific T cells was detected in 8/24 patients (33%) when stimulated with three master pools: pool 4 (*n* = 4; median: control = 0.025%, patients = 0.173%), pool 5 (*n* = 3; median: control = 0.005%, patients = 0.111%) and pool 10 (*n* = 4; median: control = 0.003%, patients = 0.029%; Figure 1a). D2R‐specific T cells were not detected in controls (0/16; Figure 1a). The immunodominant regions of D2R were restricted to master pools 4, 5 and 10 as CD4^+^ T‐cell activation was not observed in patients and controls in the remainder seven master pools (Supplementary figure 1). While most D2R‐specific T‐cell‐positive patients recognised one of the three immunodominant master pools (6/8, 75%), patient 2 (P2) recognised two master pools, pools 4 and 10, and P8 recognised all three master pools. The overall percentage of CD3^+^CD4^+^ T cells across all conditions tested did not vary significantly between patients (median = 44.65%; IQR = 34.08–47.30%) and controls (median = 40.35%; IQR = 38.87–45.19%; Mann–Whitney *U*‐tests, *P* = 0.86; Figure 1b). The percentage of T‐cell response against D2R in positive patients (median = 0.11%; IQR = 0.07–0.15%) was not significantly different to the percentage of T‐cell response against the recall antigen, TT, in all patients (median = 0.15%; IQR = 0.03–0.40%) and controls (median = 0.17%; IQR = 0.04–1.02%; Kruskal–Wallis tests, *P* = 0.46; Figure  1c). The eight D2R‐specific T‐cell‐positive patients were seronegative for D2R antibodies. 4/23 patients were seropositive for D2R antibodies but negative for D2R‐specific T cells (Supplementary figure 2). Patients with D2R antibodies presented with chronic Tourette's syndrome with obsessive–compulsive disorder (OCD) and basal ganglia encephalitis.

D2R‐specific T cells were detected in 35% (6/17) of patients with neurodevelopmental aetiology and in 29% (2/7) of patients with suspected autoimmunity, and thus, most of the eight patients with D2R‐specific T cells had a neurodevelopmental aetiology (6/8; 75%; Table 1 and Figure 1d). The two patients with suspected autoimmunity were previously healthy but had acute‐onset chorea and were diagnosed with Sydenham's chorea, a disorder of presumed post‐streptococcal autoimmune aetiology (Table 1). On the other hand, in D2R‐specific T‐cell‐positive patients with neurodevelopmental origins, Tourette's syndrome was the dominant manifestation (6/6) and most co‐presented with obsessive–compulsive disorder (OCD, 5/6; Table 1). Their disease course was chronic, and disease duration ranged from 2 to 12 years before sampling (median = 6.5 years; Table 1). Master pools 4 and 10 induced a T‐cell response in both clinical groups; however, master pool 5 was exclusively recognised by patients with a neurodevelopmental aetiology (Figure  1d). There were no discernible clinical differences in patients who were positive or negative for D2R‐specific T‐cell activation.

**Table 1:** Clinical data of dopamine‐2 receptor‐specific T‐cell‐positive patients

Patient #	1	2	3	4	5	6	7	8
Gender	Male	Male	Female	Female	Female	Male	Male	Male
Disease duration at sampling (years)	0.17	0.04	11	2	12	5	2	8
Aetiology	Autoimmune	Autoimmune	ND	ND	ND	ND	ND	ND
Infection‐provoked onset and/or worsening	Yes	Yes	Yes	Yes	No	No	Yes	No
TS	No	No	Yes	Yes	Yes	Yes	Yes	Yes
OCD	No	No	Yes	Yes	Yes	Yes	No	Yes
Other features	Chorea	Chorea	Anorexia	Autism, ADHD, ODD	Autism, depression	‐	‐	Anxiety
Disease course at time of sampling	Acute	Acute	Chronic	Chronic	Chronic	Chronic	Chronic	Chronic

ADHD, attention‐deficit/hyperactivity disorder; ND, neurodevelopmental; OCD, obsessive–compulsive disorder; ODD, oppositional defiant disorder; TS, Tourette's syndrome

Having identified the putative regions of D2R that elicit CD4^+^ T‐cell activation, the immunodominant regions were further delineated by deconvoluting the master pools 4, 5 and 10 (Supplementary tables 1 and 2). These D2R sub‐pools were cultured with the peripheral blood mononuclear cells (PBMCs) of 7/8 D2R‐specific T‐cell‐positive patients to determine the relative activation of CD4^+^CD25^+^CD134^+^ T cells by each sub‐pool. P2, P3 and P5, who were previously shown to recognise master pool 4, demonstrated a preferential activation to sub‐pool 4A (P2: 61.5%, P3: 100% and P5: 100%) over pool 4B (Figure 1e). Master pool 5 induced CD4^+^ T‐cell activation in P4 and P6, and in particular, sub‐pool 5B (P4: 100%, P6: 53.9%) elicited a greater proportion of T‐cell activation than sub‐pool 5A (P4: 0.00%, P6: 46.1%; Figure  1e). T cells in P1, P2 and P7 were activated in response to master pool 10. Sub‐pool 10B was the only pool that T cells in P1 and P2 recognised (100%), while in P7, sub‐pool 10A and 10B equally stimulated T‐cell activation (50%; Figure 1e). Sub‐pool 10C was not tested in P7. Overall, a greater activation response to sub‐pools 4A, 5B and 10B suggested that they encompassed the T‐cell immunodominant regions on D2R. These three regions corresponded to amino acid (aa)121–131, aa171–181 and aa396–416, respectively, and are localised to the second intracellular loop, second and third extracellular loops and the seventh transmembrane domain (Figure 1f).

### Presentation of immunodominant D2R peptides was restricted by HLA‐D molecules

**Figure 2:**
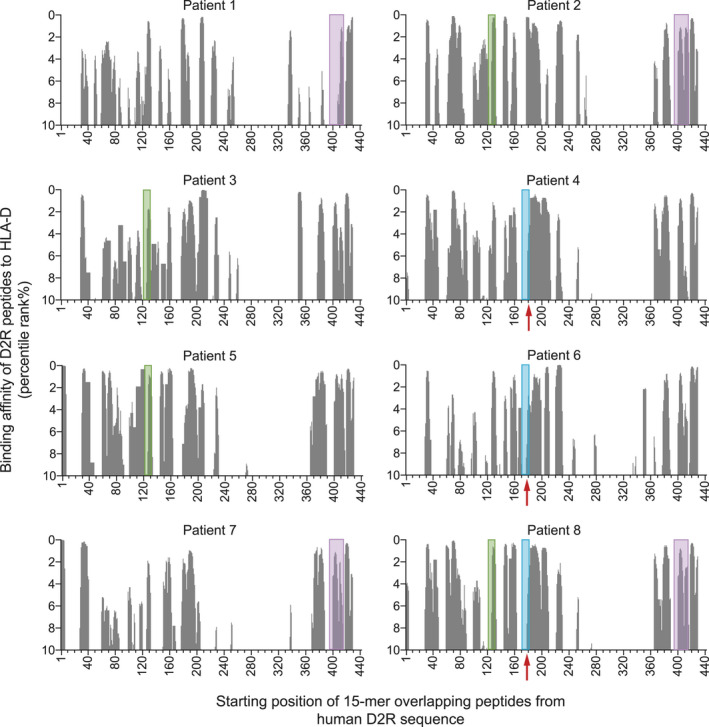
Immunodominant dopamine‐2 receptor (D2R) peptides identified *in vitro* were high‐affinity binders of HLA‐D molecules. In D2R‐specific T‐cell‐positive patients (*n* = 8), the likelihood of HLA‐DRB1, HLA‐DQA1, HLA‐DQB1, HLA‐DPA1 and HLA‐DPB1 alleles to present D2R 15‐mer peptides encompassing the full‐length protein was predicted using the Immune Epitope Database (IEDB). The top 10 percentile ranks are shown on the y‐axis and represent the binding affinity of D2R peptides to HLA‐D. A lower percentile rank is indicative of a higher binding affinity to a HLA‐D molecule. Shaded areas denote the peptide pool which elicited CD4^+^T‐cell activation in that patient using the CD25/CD134 assay (green = peptide pool 4A; blue = peptide pool 5B; purple = peptide pool 10B; red arrow indicates peptides within blue shaded area).

CD4^+^ T‐cell recognition and activation by an antigen require a robust binding of the peptide to the HLA class II molecule, HLA‐D, on antigen‐presenting cells. Using the immune epitope database (IEDB), we predicted the likelihood of the D2R peptides within the immunodominant pools to bind to the HLA‐D genotype of D2R‐specific T‐cell‐positive patients (*n* = 8; Supplementary table 3). Clusters of 15‐mer peptides across the D2R protein sequence had prediction ranks in the top 10 percentile, indicating a high binding affinity to HLA‐D, and these clusters included peptides from the immunodominant pools which elicited T‐cell activation *in vitro* (Figure 2). The majority of peptides of sub‐pool 4A and sub‐pool 10B sequence bound with high affinity (median percentile rank = 1.55 and 2.6, respectively) to HLA‐D. Peptides of sub‐pool 5B sequence were also predicted to be high binders, but to a lesser extent (median percentile rank = 4). In P8, T‐cell activation was elicited by master pools 4, 5 and 10 (Figure 1a), but the precise sub‐pool that induced activation within the respective master pool could not be determined *in vitro*. Nevertheless, peptides of sub‐pools 4A, 5B and 10B were predicted to bind with high affinity to this patient's HLA genotype (Figure 2). Computational predictions of T‐cell epitopes agreed with our findings from *in vitro* studies, further supporting these three regions as the likely T‐cell immunodominant regions of D2R.

In addition to recognising the peptide of an HLA‐peptide complex, T cells must also recognise the specific HLA variant in the complex for full activation. Hence, certain HLA genotypes may predispose a patient to D2R‐specific T‐cell activation. HLA‐DPA1*01:03:01 and HLA‐DPB1*04:01:01 were notably over‐represented in D2R‐specific T‐cell‐positive patients (Supplementary table 4). These two genotypes were prevalent in 6/8 (75%) patients and, moreover, co‐existed in a patient, suggesting that both genotypes may play a contributing role (Supplementary tables 3 and 4). There was also a higher occurrence of HLA‐DQA1*01:02:01 (3/8; 37.5%) and HLA‐DQB1*03:01:01 (3/8; 37.5%; Supplementary table 4).

### D2R‐specific T‐cell activation in patients with movement and psychiatric disorders was associated with a pro‐inflammatory cytokine secretion profile

**Figure 3:**
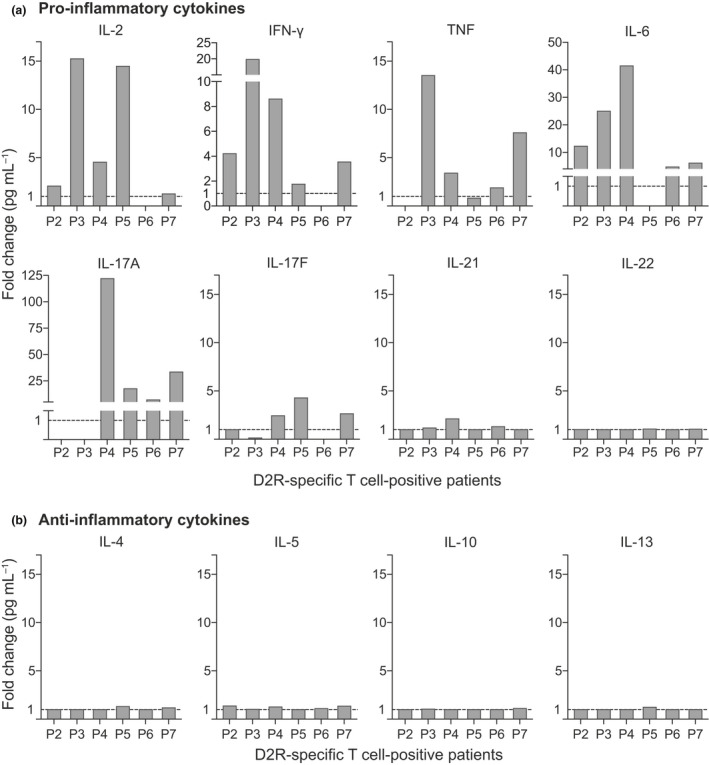
Dopamine‐2 receptor (D2R)‐specific T‐cell‐positive patients exhibited a pro‐inflammatory cytokine profile. Peripheral blood mononuclear cells of D2R‐specific T‐cell‐positive patients (*n* = 6) were stimulated with immunodominant sub‐pools 4, 5 and 10. The supernatant of each sub‐pool stimulation was tested once in duplicates on a multiplex cytometric bead array and IFN‐γ ELISA to characterise the cytokine secretions. Compared to the controls (dashed line = normalised median of controls), D2R‐specific T‐cell‐positive patients had **(a)** elevated levels of pro‐inflammatory cytokines IL‐2 (4/6), IFN‐γ (1/3), TNF (4/6), IL‐6 (5/6), IL‐17A (4/6) and IL‐17F (3/6), and slightly higher IL‐21 (1/6). **(b)** Contrastingly, D2R‐specific T‐cell‐positive patients had similar concentration of Th2 cytokines (6/6) as the controls.

**Figure 4:**
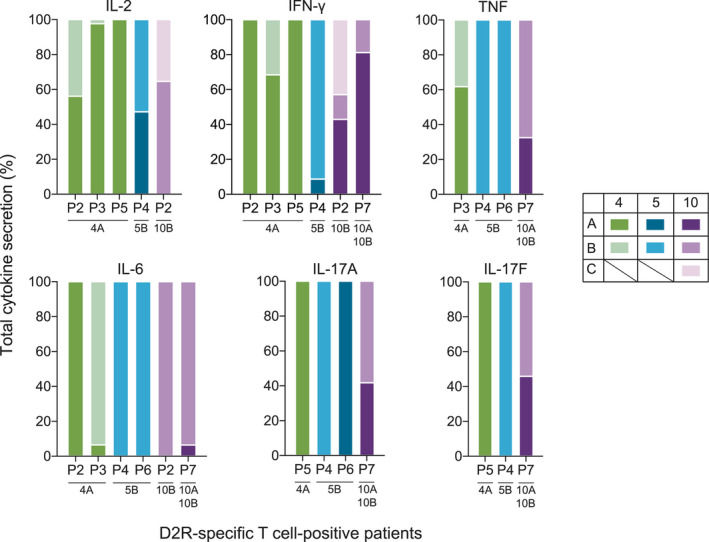
An elevated pro‐inflammatory cytokine secretion was associated with the dopamine‐2 receptor (D2R) peptide sub‐pools which also activated D2R‐specific T cells. Peripheral blood mononuclear cells (PBMCs) from D2R‐specific T‐cell‐positive patients (*n* = 6) were stimulated with the sub‐pools of immunodominant master pools 4, 5 and 10. The supernatant of each sub‐pool stimulation was tested in duplicates on a multiplex cytometric bead array and IFN‐γ ELISA to characterise the cytokine secretions. Elevated levels of pro‐inflammatory cytokines in each patient were found to be predominantly secreted when the PBMCs were stimulated by D2R sub‐pools 4A, 5B and 10B as compared to the other sub‐pools 4B, 5A, 10A and 10C. These sub‐pools that induced these increased cytokine secretions largely corresponded to the sub‐pools that also elicited activation of D2R‐specific T cells, as specified beneath each patient.

A hallmark of activated and functional T cells is the secretion of cytokines which modulate the inflammatory response. As elevated pro‐inflammatory cytokines were reported in other neuroinflammatory diseases,^33^, ^34^, ^40^, ^41^, ^42^ we determined whether D2R‐specific T‐cell activation corresponded with an increase in pro‐inflammatory cytokines. We characterised the cytokine milieu of the supernatant of PBMCs stimulated with the D2R sub‐pools of immunodominant master pools 4, 5 and 10. When the combined cytokine response to all sub‐pools in patients was compared to the normalised median of controls, pro‐inflammatory cytokine characteristics of T helper 1 (Th1) and T helper 17 (Th17) cells were notably elevated in 6/6 D2R‐specific T‐cell‐positive patients (Figure 3a). In particular, IL‐6 was 4‐ to 41‐fold higher in 5/6 patients and IL‐17A was 7‐ to 122‐fold higher in 4/6 patients than controls (*n* = 11). Sub‐pools of D2R also induced increased secretion of IL‐2 (4/6), IFN‐γ (5/6), TNF (4/6) and, to a lesser extent, IL‐17F (3/6; Figure  3a). Conversely, the concentration of anti‐inflammatory T helper 2 (Th2) cytokines IL‐4, IL‐5, IL‐10 and IL‐13 was consistently low in all D2R‐specific T‐cell‐positive patients and similar to controls (Figure 3b). Further analysis showed that elevated secretion of these six pro‐inflammatory cytokines was predominantly induced by sub‐pools 4A, 5B and 10B (Figure 4). Moreover, in each patient, increased cytokine secretion was associated with the sub‐pool, which induced D2R‐specific T‐cell activation (Figure 1c and Figure 4).

## Discussion

It is widely accepted that there is a pathogenic inflammatory response in movement and psychiatric disorders, such as NMDAR antibody‐associated encephalitis and Sydenham's chorea.^1^, ^2^, ^3^, ^4^, ^5^, ^6^, ^7^ Additionally, there is an emerging role of the immune system in neurodevelopmental disorders such as Tourette's syndrome and associated neuropsychiatric disorders.^43^ Given these compelling findings, we aimed to study the immune dysfunction in paediatric patients with a range of acute and chronic movement and psychiatric disorders of autoimmune and neurodevelopmental aetiology, and a promising target of immune attack in these disorders is D2R.^12^, ^13^, ^14^, ^15^, ^16^, ^17^, ^18^, ^19^ Herein, we identified and characterised autoreactive D2R‐specific T cells in a subset of paediatric patients with movement and psychiatric disorders. Three discrete immunodominant regions of D2R induced CD4^+^ T‐cell activation, corresponding to aa121–131, aa171–181 and aa396–416 that are localised on the second intracellular loop, second and third extracellular loops and the seventh transmembrane domain. These regions were predicted to bind with high affinity to the HLA‐D genotype of D2R‐specific T‐cell‐positive patients, and T‐cell activation positively correlated with elevated levels of pro‐inflammatory cytokines. Together, these findings support a role for autoreactive T cells in the pathogenesis of a subgroup of movement and psychiatric disorders.

In this single‐centre study, D2R‐specific T cells were detected in patients with a variety of acute and chronic movement and psychiatric disorders. Acute chorea was the most common presentation in D2R‐specific T‐cell‐positive patients with probable autoimmunity. On the other hand, D2R‐specific T‐cell‐positive patients with neurodevelopmental disorders had a chronic disease course and Tourette's syndrome concomitant with OCD was the prominent feature. This result supports the existing paradigm of dopamine dysregulation in Tourette's syndrome^17^, ^44^, ^45^ and is consistent with the recent report of altered frequencies in functional subsets of circulating CD4^+^ T cells in paediatric patients with OCD.^46^ While PANS was a common feature in our cohort and recent findings have suggested neuroinflammatory involvement,^25^ D2R‐specific T cells were not observed in these patients. D2R‐specific T cells were identified in both an acute course and chronic course of movement and psychiatric disorders, suggesting different modes of T‐cell activity in different disease courses. Activated D2R‐specific T cells in acute autoimmune movement and psychiatric disorders suggest a recent encounter with its cognate antigen. In chronic neurodevelopmental disorders, however, activated D2R‐specific T cells may reflect a persistently active immune system. Interestingly, D2R‐specific T cells in patients with neurodevelopmental disorders recognised more immunodominant regions of D2R than in patients with autoimmune movement and psychiatric disorders. While there were more patients with neurodevelopmental disorders, this difference may represent epitope spreading in the chronic course of neurodevelopmental disorders. Although we did not analyse serial samples, Vaknin‐Debminsky and colleagues have demonstrated in a longitudinal study that T cells in patients with NMOSD gradually recognised a broader range of aquaporin‐4 (AQP4) peptides.^33^ A similar expansion in autoreactive T‐cell antigen specificity over time has been observed in MS and type 1 diabetes.^47^, ^48^

Dopamine‐2 receptor has been previously described as an immune target of antibodies. However, patients positive for D2R‐specific T cells were all seronegative for D2R antibodies. This finding adds to the growing reports of neuroimmune encephalitis cases that are negative for known neuronal antibodies, but are responsive to immunotherapy.^49^, ^50^ D2R antibodies can be detected with methodologies such as an ELISA^13^, ^15^, ^16^, ^22^, ^25^; however, the live cell‐based assay used in this study is a widely accepted method for neuronal antibody detection. Thus, the detection of D2R‐specific T cells may define a subset of movement and psychiatric disorders. Conversely, D2R antibody seropositivity in our cohort was not associated with activated D2R‐specific T cells. Despite D2R antibodies being IgG,^17^ an antibody isotype dependent on interactions with CD4^+^ T follicular helper (Tfh) cells, the absence of D2R‐specific CD4^+^ T cells in these patients can be attributed to certain factors. Unlike antibodies, which are readily accessible in the peripheral blood, Tfh cells predominantly reside in secondary lymph nodes.^51^, ^52^ Hence, the time of peripheral blood sampling can affect the detection of antigen‐specific CD4^+^ T cells. This factor is compounded by the rarity of antigen‐specific T cells in the peripheral blood^53^ and challenges the sensitivity of a detection test. Nevertheless, the CD25/CD134 assay that we utilised has been shown to be more sensitive and specific than other T‐cell detection methods in identifying rare antigen‐specific memory T cells.^39^, ^54^, ^55^

T‐cell autoreactivity against D2R demonstrated by the CD25/CD134 assay was corroborated by computational predictions of HLA binding to D2R peptides. The three D2R immunodominant regions identified in the *in vitro* assay were predicted to bind with high affinity to the HLA‐D genotype of patients who harboured D2R‐specific T cells. This finding underscores the immunogenicity of these immunodominant regions, as a complete activation of antigen‐specific T cells requires recognition of the entire HLA–peptide complex, and further supports the immune dysregulation hypothesis. It was notable that HLA‐DPA1*01:03:01 and HLA‐DPB1*04:01:01 were over‐represented in patients with D2R‐specific T cells and demonstrated haplotypic association. These two alleles were prevalent in Polynesian and Amerindian populations, and HLA‐DPB1*04:01:01 was also observed in some oriental and Caucasoid groups.^56^, ^57^, ^58^, ^59^, ^60^, ^61^ In comparison, our cohort consisted of a mixed population who were not of Polynesian or Amerindian background. Specific HLA alleles and linked haplotypes have been strongly associated with other neurological neuroimmune diseases such as leucine‐rich glioma‐inactivated 1 (LGI1), contactin‐associated protein‐like 2 (CASPR2) and *N*‐methyl‐d‐aspartic acid receptor (NMDAR) antibody‐associated encephalitis.^62^, ^63^, ^64^ The prevalent HLA alleles in these disorders were HLA‐DRB1*07 linked to HLA‐DRB4, HLA‐DRB1*11:01 and HLA‐DRB1*16:02, respectively. Variations in HLA associations across these encephalitides and in patients with D2R‐specific T cells highlight the contribution of different genetic factors to the genetic susceptibility of these conditions. Strong genetic associations cannot be established in our small cohort of D2R‐specific T‐cell‐positive patients, but the frequent occurrence of these particular alleles in our patients warrants future large‐scale studies.

The pro‐inflammatory cytokine profile in patients with activated D2R‐specific T cells suggested that these cells exhibited a Th1 and Th17 bias. There was a pronounced increase in IL‐2, IFN‐ γ, TNF, IL‐6, IL‐17A and IL‐17F in D2R‐specific T‐cell‐positive patients, while an anti‐inflammatory cytokine secretion was comparable to controls. While these pro‐inflammatory cytokines can be secreted by different immune cells, they are characteristic of Th1 and Th17 cells and their elevated levels were associated with D2R‐specific T‐cell activation. Although we studied the peripheral response of D2R‐specific T cells, Th1 and Th17 signature cytokines have been reported to be the markers of intrathecal inflammation.^41^ Th1 and Th17 cells and their associated cytokines have been strongly implicated in a range of neuroinflammatory diseases, including NMDAR antibody‐associated encephalitis and demyelinating disorders such as MS, NMOSD and acute disseminated encephalomyelitis.^33^, ^34^, ^40^, ^41^, ^65^ Despite the varied phenomenology, neuroimmune encephalitis and demyelinating disorders both positively correlated with Th1 and Th17 cytokines, and the cytokine profile of these immune‐mediated aetiologies differed from an infectious aetiology.^41^, ^66^ These pro‐inflammatory cytokines can contribute to neuroinflammation by various mechanisms including breakdown of the blood–brain barrier (BBB) and attracting other immune cells, such as neutrophils and macrophages.^27^, ^67^

In addition to a role in neuroinflammation, it is plausible that D2R‐specific T cells can interfere with immune system functions as dopamine receptors are widely expressed by immune cells.^10^, ^11^, ^68^, ^69^, ^70^, ^71^ Dopaminergic signalling can modulate immunological processes, such as T‐ and B‐cell interactions in antibody production, activation of naïve T cells, inhibition of stimulated T cells, suppression of regulatory T cells, cytokine secretion, and cellular trafficking and chemotactic migration of T cells.^10^, ^11^, ^72^, ^73^, ^74^ Some studies have reported abnormalities in the expression of dopamine receptors on immune cells or impaired immune function in neurological and psychiatric disorders, including schizophrenia, Parkinson's disease and Tourette's syndrome.^75^, ^76^, ^77^, ^78^, ^79^ It can then be hypothesised that patients in this study may have abnormal D2R expression on immune cells or that autoreactive D2R‐specific T cells may target immune cells, which express, process and present dopamine receptors. These factors can contribute to a defective immune system and subsequent deleterious effects on the CNS.

The mechanisms that give rise to autoreactive T cells remain an important, yet unanswered question. Molecular mimicry has often been proposed and theorises that cross‐reactivity between microbial structures and host proteins results in an immune response misdirected to the host. This theory has been supported by the evidence that there was 90% homology between the T‐cell epitope of AQP4 and the bacterium *Clostridium perfringens*,^34^ and the induction of a Th17‐biased response in mice when infected with *Clostridium*.^80^ A similar phenomenon has been described wherein a D2R antibody epitope shared a sequence with an unknown protein of *Penicillium*,^19^ and a streptococcal infection led to the production of cross‐reactive autoantibodies in Sydenham's chorea.^13^, ^81^, ^82^ Sequence homology between T‐cell immunodominant regions of D2R and microbial structures was not observed in this study (data not shown). Some D2R‐specific T‐cell‐positive patients, however, exhibited disease exacerbation upon infection, suggesting that an activated immune response could erroneously target self‐proteins as a bystander effect of fighting an infection.^25^, ^83^ This notion has been demonstrated in a murine model wherein repeated infections led to the expansion and migration of Th17 cells from the peripheral lymph nodes to the brain.^26^ Once in the brain, these reactive T cells induced secretion of IL‐17A, activation of microglia and breakdown of the BBB, which could augment neuroinflammation. Additionally, an impaired innate immune response may contribute to autoimmunity. For instance, monocytes in NMOSD patients had elevated IL‐6 secretion and expression of co‐stimulatory molecules that could perpetuate a Th17 response.^34^ An activated complement system and microglia have also been increasingly recognised for their role in neuroinflammation in diseases such as MS, Tourette's syndrome, Parkinson's disease and schizophrenia.^84^, ^85^, ^86^, ^87^, ^88^ Our study explored CD4^+^ helper T cells; however, CD8^+^ cytotoxic T cells have been implicated in the pathophysiology of MS, Rasmussen's encephalitis and paraneoplastic syndromes.^29^, ^30^, ^31^, ^35^, ^36^, ^37^, ^38^ Likewise, CD8^+^ T cells may also have a contributing role in D2R‐specific T‐cell autoimmunity through activation and subsequent secretion of pro‐inflammatory cytokines. Given these observations, future studies are needed to evaluate the role of innate and cytotoxic T‐cell immune dysfunction in neuroimmune movement and psychiatric disorders.

Identifying and characterising the neuroinflammation associated with a reactive T‐cell response offer novel alternatives for the treatment of movement and psychiatric disorders. Strategies may include inhibiting T‐cell proliferation, restricting T‐cell trafficking or targeting cytokines produced by T cells.^89^, ^90^ An example of the latter is tocilizumab, a monoclonal antibody that targets the receptor of IL‐6, a cytokine notably elevated in patients positive for D2R‐specific T cells. Interestingly, tocilizumab has been effective in the treatment of neuroimmune encephalitis and NMOSD patients unresponsive to standard immunotherapies.^91^, ^92^, ^93^, ^94^ The co‐stimulatory molecule CD134 on activated T cells is another viable treatment target. Blockade of CD134 with a monoclonal antibody decreased mononuclear cell infiltration into the spinal cord of experimental autoimmune encephalomyelitis mouse models and reduced pro‐inflammatory response in a murine model of rheumatoid arthritis.^95^, ^96^ Further studies are needed to assess the implications of these results on patient treatments.

In summary, we have identified and characterised pro‐inflammatory D2R‐specific T cells in patients with movement and psychiatric disorders who are negative for D2R antibodies. Notably, these T cells are associated with a Th1 and Th17 phenotype, suggesting a T‐cell‐driven immune response. This suggests that autoreactive D2R‐specific T cells may be a hallmark of a subgroup of movement and psychiatric disorders from both an autoimmune origin and neurodevelopmental origin and prompts further investigation into their pathogenic role. Further knowledge can aid in discriminating patients who are phenotypically similar and consequently encourage treatment regimes towards T‐cell‐directed therapies for improved patient outcomes.

## Methods

### Subjects

This study investigated paediatric patients (*n* = 24) seen from 2016 to 2019 at The Children's Hospital at Westmead, Sydney, Australia. Patients presented with a range of movement and psychiatric disorders that were of suspected autoimmune aetiology (*n* = 7)^43^ or neurodevelopmental origin (*n* = 17)^97^ (Table 2). All patients were symptomatic at the time of blood sampling and were not on immunotherapy. Patients with Sydenham's chorea (*n* = 5) fulfilled the revised Jones criteria of acute rheumatic fever and the criteria defined by Cardoso *et al*.^98^, ^99^ All patients with Tourette's syndrome (*n* = 17) fulfilled the DSM‐IV criteria for this disorder. All patients with PANS (*n* = 7) fulfilled the criteria developed by Chang *et al*.^100^ The control cohort (*n* = 16; seven males; median age = 11.5 years; range = 6–16 years) consisted of children with neurological disorders (*n* = 7; 4/7 confirmed genetic, 3/7 suspected genetic brain disease), children investigated for growth failure (*n* = 8) and a child with coeliac disease (*n* = 1). The ethics approval for this study (NEAF 12/SCHN/395) was granted by the human research ethics committees of the Sydney Children's Hospital Network. Written informed consent was obtained from all participants or their carers.

**Table 2:** Summary of demographics and clinical characteristics of patients with movement and psychiatric disorders classified by aetiology

	Autoimmune	Neurodevelopmental
Number of participants	7^a^	17
Male:female	3:4	9:8
Median age at sampling (range; years)	12 (10–17)	11 (3–16)
Median disease duration at sampling (range; years)	0.17 (0.04–12)	3 (0.04–12)
Clinical characteristics		
Tourette's syndrome only, *n*	0	2
Tourette's syndrome and OCD, *n*	0	15^b^
Sydenham's chorea	5	0
Other neuropsychiatric features and movement disorders, *n*	Basal ganglia encephalitis 2	Anorexia 1; anxiety 4; autism 4; ADHD 2; depression 4; fatigue 1; mood 1; ODD 1; sensory 1
Course of disease, *n*		
Acute	6	1
Chronic	1	16

ADHD, attention‐deficit/hyperactivity disorder; OCD, obsessive–compulsive disorder; ODD, oppositional defiant disorder

^a^ The seven autoimmune patients fulfilled the criteria for Sydenham's chorea (*n* = 5) according to the revised Jones criteria of acute rheumatic fever and the criteria by Cardoso *et al*.^98^, ^99^ or basal ganglia encephalitis (*n* = 2) with specific basal ganglia inflammation, as described.^17^

^b^ Seven patients with Tourette's syndrome had acute infection‐provoked onset consistent with paediatric acute‐onset neuropsychiatric syndrome.

### Peptides and antigens

A library of 87 peptides were synthesised based on the complete sequence of the human D2R protein (Mimotopes, Australia; Supplementary table 1). Each 15‐mer peptide overlapped by 10 amino acids and had > 85% purity. Excluding 20 peptides that were insoluble mainly because of their hydrophobicity, the peptides were sequentially grouped into 10 master pools for whole blood cultures (Supplementary table 1). Select master pools were further split into sub‐pools of two or three peptides for PBMC cultures (Supplementary table 2). Each peptide was used at a final concentration of 9 μg mL^−1^. Cells were stimulated with two positive control antigens: 1 μg mL^−1^ of staphylococcus enterotoxin B (SEB; Sigma‐Aldrich, St Louis, MO, USA), a super‐antigen which binds to a subset of T cells in a non‐antigen‐specific manner and induces substantial activation and proliferation, and 2 μg mL^−1^ of tetanus toxoid (TT; Enzo Life Sciences, Farmingdale, NY, USA), a recall antigen which requires processing and presentation by antigen‐presenting cells in association with HLA class II molecules to induce activation of TT‐specific T cells via the T‐cell receptor.

### CD25/CD134 assay for the detection of D2R‐specific T cells

To identify activated D2R‐specific CD4^+^ T cells, blood was collected concurrently for CD25/CD134 assay with whole blood^39^, ^101^ and PBMCs. PBMCs were isolated by density gradient centrifugation using the Ficoll‐Paque PLUS (GE Healthcare Lifesciences, Chicago, IL, USA).

Fresh sodium heparinised whole blood was diluted with Iscove's Modified Dulbecco's Media (IMDM; 1:1; Gibco, Gaithersburg, MD, USA) and cultured in sterile 24‐well tissue culture plate with media alone (unstimulated), positive control antigens (SEB and TT) or D2R peptide master pools. In four patients with limited sample, D2R peptide master pool 1 and pool 7 were not tested as they were previously shown not to elicit a T‐cell activation. Similarly, cryopreserved PBMCs were cultured in 96‐well U‐bottom tissue culture plates at 5 × 10^5^ cells well^−1^ (200 μL well^−1^) in RPMI (Gibco) supplemented with 10% heat‐inactivated human AB serum (Sigma‐Aldrich), penicillin and streptomycin. Each PBMC sample was cultured with complete media alone, TT and D2R sub‐pools of the master pools previously shown to elicit T‐cell activation in the whole blood CD25/CD134 assay. Whole blood and PBMC cultures were incubated for 42–44 h at 37°C in a humidified atmosphere of 5% CO_2_.

After 42–44 h of culture, 200 μL of whole blood or PBMC cultures was stained with mouse anti‐human CD3‐V450, CD4‐FITC, CD25‐APC and CD134‐PE (BD Biosciences, San Jose, CA, USA) for 15 min at room temperature (Supplementary table 5). Red blood cells in whole blood samples were lysed with BD Pharm Lyse (BD Biosciences), as per the manufacturer's protocol. Cells were acquired on a five‐laser BD LSR II Flow Cytometer (BD Biosciences). Data were analysed with FlowJo 10.4.1 (TreeStar, FLowJoTM Software, Ashland, OR, USA), Excel (Microsoft, Redmond, WA, USA) and GraphPad Prism (GraphPad Software, San Diego, CA, USA). CD3^+^CD4^+^ helper T cells were analysed for co‐expression of CD134 and high CD25 based on the negative (unstimulated) and positive (SEB and TT) controls included in each experiment (Supplementary figure 3). A minimum of 40 000 and 30 000 lymphocytes were analysed for the whole blood and PBMC cultures, respectively. The signal of the unstimulated condition was subtracted from the antigen‐stimulated conditions to remove background. In whole blood cultures, a positive D2R‐specific T‐cell activation was established if the frequency of CD25^+^CD134^+^CD4^+^ T cells exceeded a threshold set by the mean + 3SD of the control cohort. One control was excluded from the control cohort of pools 1, 4 and 10 as they were an outlier as per the Grubbs test.

### HLA genotyping and HLA‐D2R peptide‐binding prediction

HLA class II molecules are crucial in presenting an antigen to CD4^+^ T cells. The genes encoding HLA class II proteins are highly polymorphic and, unsurprisingly, have been commonly associated with autoreactive CD4^+^ T cells. Given this, HLA class II genotyping was performed in patients shown to harbour D2R‐specific T cells (*n* = 8) using the AlloSeq Tx assay (CareDx, Brisbane, CA, USA). HLA alleles were fully resolved to the second or third field as defined by the IPD‐IMGT/HLA database (http://hla.alleles.org/nomenclature/naming.html).

The online server tool MHC‐II prediction from the Immune Epitope Database (IEDB; http://tools.iedb.org/mhcii/) was used to assess the binding prediction of D2R peptides to class II HLA alleles of each patient. With the exception of new alleles or one allele not documented in IEDB, all possible allele combinations for each class and both chromosomes were examined, resulting in two combinations for HLA‐DR and four for HLA‐DQ and HLA‐DP. The sequence of the full protein was inputted and analysed as 15‐mer peptides overlapping by 10 amino acids. Peptides were considered predicted binders if the consensus percentile rank was < 10%.^102^

### Cytokine secretion profiling

Following 42–44 h of stimulation, the supernatant of the PBMC cultures was collected and frozen at −80°C for later use. The supernatant of six D2R‐specific T‐cell‐positive patients was analysed for secreted cytokines with the LEGENDPlex Human Th Cytokine Panel 13‐plex Kit (BioLegend, San Diego, CA, USA) and an IFN‐γ enzyme‐linked immunosorbent assay (ELISA; Mabtech Stockholm, Sweden) used as per the manufacturer's protocol. The following cytokines were quantified: IL‐2, IL‐4, IL‐5, IL‐6, IL‐9, IL‐10, IL‐13, IL‐17A, IL‐17F, IL‐21, IL‐22, IFN‐γ and TNF. All samples were measured undiluted and acquired on BD LSR II and BD FACSCanto II. Data were analysed with LEGENDPlex Data Analysis Software v8 (BioLegend), Excel and GraphPad Prism.

### Flow cytometry live cell‐based assay for the detection of D2R antibodies

A flow cytometry live cell‐based assay was used to detect the binding of patient serum antibodies against surface human D2R, an accepted method of neuronal antibody detection,^4^ and was performed as previously described.^17^, ^18^, ^19^, ^21^ Briefly, transfected live HEK293 cells expressing D2R were incubated with serum (1:50),^103^ followed by staining with Alexa Fluor 647‐conjugated anti‐human IgG (H + L; Thermo Fisher Scientific, Waltham, MA, USA). Cells were acquired live using the high‐throughput system on a five‐laser BD LSR II Flow Cytometer and analysed with FlowJo 10.4.1, Excel and GraphPad Prism. The threshold was calculated as the mean + 3SD of the control cohort, and a patient was reported positive for D2R antibodies if they were above this threshold in at least two of three independent experiments. Only 23 of 24 patients were tested as the serum of one patient was not available. Antibodies against other dopamine receptor subtypes were not detected in our previous study and therefore were not tested.^17^

### Statistics

Subject groups were compared with non‐parametric two‐tailed Mann–Whitney *U*‐tests or Kruskal–Wallis tests, where appropriate, using GraphPad Prism 7. Statistical significance was determined to be *P* < 0.05.

## Author contributions

**Deepti Pilli:** Conceptualization; Investigation; Methodology; Writing‐original draft; Writing‐review & editing. **Alicia Zou:** Formal analysis; Investigation; Methodology; Writing‐review & editing. **Ruebena Dawes:** Formal analysis; Investigation; Writing‐original draft. **Joseph A Lopez:** Formal analysis; Investigation; Methodology; Writing‐review & editing. **Fiona Tea:** Formal analysis; Investigation; Methodology; Writing‐review & editing. **Ganesha Liyanage:** Formal analysis; Investigation; Methodology; Writing‐review & editing. **Fiona X.Z. Lee:** Investigation; Methodology; Writing‐review & editing. **Vera Merheb:** Investigation; Methodology; Writing‐review & editing. **Samuel Houston:** Data curation; Methodology; Software; Writing‐review & editing. **Aleha Pillay:** Data curation; Formal analysis; Methodology; Writing‐review & editing. **Hannah Jones:** Formal analysis; Investigation; Writing‐review & editing. **Sudarshini Ramanathan:** Investigation; Methodology; Writing‐review & editing. **Shekeeb Mohammad:** Data curation; Formal analysis; Investigation; Writing‐review & editing. **Anthony D Kelleher:** Formal analysis; Investigation; Methodology; Writing‐review & editing. **Stephen I. Alexander:** Formal analysis; Investigation; Methodology; Writing‐review & editing. **Russell C Dale:** Formal analysis; Investigation; Writing‐review & editing. **Fabienne Brilot:** Conceptualization; Formal analysis; Funding acquisition; Investigation; Methodology; Supervision; Writing‐review & editing.

## Conflict of interest

DP reports funding from the Neville Brown Scholarship (Australia). SR reports fellowship research funding from the National Health and Medical Research Council (Australia). RCD and FB have received research funding from the Trish Multiple Sclerosis Research Foundation, Multiple Sclerosis Research Australia, the Petre Foundation and the National Health Medical Research Council (Australia). They have received honoraria from Biogen Idec and Merck Serono as invited speakers. AZ, RD, JAL, FT, GL, FXZL, VM, SDH, AP, HFJ, SM, ADK and SIA declare no competing interests.
