# Supplementary material for: Pro‐inflammatory dopamine‐2 receptor‐specific T cells in paediatric movement and psychiatric disorders
Source: Clin Transl Immunology. 2020 Dec 17;9(12):e1229. doi: 10.1002/cti2.1229 (PMC7780098; doi:10.1002/cti2.1229)
Supplement: Supplementary file 1 [file CTI2-9-e1229-s001.docx]

# Supplementary Information

## Supplementary Tables

**Supplementary table 1.** A library of overlapping human D2R peptides were sequentially grouped into ten master peptide pools for high throughput screening of T cell immunodominant regions.

| **Pool** | **Peptide** | **AA** | **Sequence** | **Pool** | **Peptide** | **AA** | **Sequence** |
| --- | --- | --- | --- | --- | --- | --- | --- |
| **1** | 1 | 1-15 | MDPLNLSWYDDDLER | **5**  (cont.) | 36 | 175-190 | NADQNECIIANPAFV |
|  | 2 | 6-20 | LSWYDDDLERQNWSR |  | 37 | 181-195 | ECIIANPAFVVYSSI |
|  | 3 | 11-25 | DDLERQNWSRPFNGS | **6** | 42 | 206-220 | LLVYIKIYIVLRRRR |
|  | 4 | 26-30 | QNWSRPFNGSDGKAD |  | 43 | 211-225 | KIYIVLRRRRKRVNT |
|  | 5 | 21-35 | PFNGSDGKADRPHYN |  | 44 | 216-230 | LRRRRKRVNTKRSSR |
|  | 6 | 26-40 | DGKADRPHYNYYATL |  | 45 | 221-235 | KRVNTKRSSRAFRAH |
|  | 7 | 31-45 | RPHYNYYATLLTLLI |  | 46 | 226-240 | KRSSRAFRAHLRAPL |
| **2** | 11 | 51-65 | GNVLVCMAVSREKAL |  | 47 | 213-245 | AFRAHLRAPLKGNCT |
|  | 12 | 56-70 | CMAVSREKALQTTTN |  | 48 | 236-250 | LRAPLKGNCTHPEDM |
|  | 13 | 61-75 | REKALQTTTNYLIVS |  | 49 | 241-255 | KGNCTHPEDMKLCTV |
| **3** | 17 | 81-95 | LLVATLVMPWVVYLE |  | 50 | 246-260 | HPEDMKLCTVIMKSN |
|  | 19 | 95-105 | VVYLEVVGEWKFSRI |  | 51 | 251-265 | KLCTVIMKSNGSFPV |
|  | 20 | 96-110 | VVGEWKFSRIHCDIF | **7** | 52 | 256-270 | IMKSNGSFPVNRRRV |
|  | 21 | 101-115 | KFSRIHCDIFVTLDV |  | 53 | 261-275 | GSFPVNRRRVEAARR |
| **4** | 25 | 121-135 | SILNLCAISIDRYTA |  | 54 | 266-280 | NRRRVEAARRAQELE |
|  | 26 | 126-140 | CAISIDRYTAVAMPM |  | 55 | 271-285 | EAARRAQELEMEMLS |
|  | 27 | 131-145 | DRYTAVAMPMLYNTR |  | 56 | 276-290 | AQELEMEMLSSTSPP |
|  | 28 | 136-150 | VAMPMLYNTRYSSKR |  | 57 | 281-295 | MEMLSSTSPPERTRY |
|  | 29 | 141-155 | LYNTRYSSKRRVTVM |  | 58 | 286-300 | STSPPERTRYSPIPP |
| **5** | 32 | 156-170 | ISIVWVLSFTISCPL |  | 59 | 291-305 | ERTRYSPIPPSHHQL |
|  | 33 | 161-175 | VLSFTISCPLLFGLN |  | 60 | 296-310 | SPIPPSHHQLTLPDP |
|  | 34 | 166-180 | ISCPLLFGLNNADQN |  | 61 | 301-315 | SHHQLTLPDPSHHGL |
|  | 35 | 171-185 | LFGLNNADQNECIIA | **8** | 62 | 306-320 | TLPDPSHHGLHSTPD |

**Supplementary table 1.** *Continued.*

| **Pool** | **Peptide** | **AA** | **Sequence** | **Pool** | **Peptide** | **AA** | **Sequence** |
| --- | --- | --- | --- | --- | --- | --- | --- |
| **8**  (cont.) | 63 | 311-325 | SHHGLHSTPDSPAKP | **10** | 77 | 381-395 | VFIICWLPFFITHIL |
|  | 64 | 316-330 | HSTPDSPAKPEKNGH |  | 78 | 386-400 | WLPFFITHILNIHCD |
|  | 65 | 321-335 | SPAKPEKNGHAKDHP |  | 79 | 391-405 | ITHILNIHCDCNIPP |
|  | 66 | 326-340 | EKNGHAKDHPKIAKI |  | 80 | 396-410 | NIHCDCNIPPVLYSA |
|  | 67 | 331-345 | AKDHPKIAKIFEIQT |  | 82 | 406-420 | VLYSAFTWLGYVNSA |
|  | 68 | 336-350 | KIAKIFEIQTMPNGK |  | 84 | 416-430 | YVNSAVNPIIYTTFN |
|  | 69 | 341-355 | FEIQTMPNGKTRTSL |  | 85 | 421-435 | VNPIIYTTFNIEFRK |
|  | 70 | 346-360 | MPNGKTRTSLKTMSR |  | 86 | 426-440 | YTTFNIEFRKAFLKI |
|  | 71 | 351-365 | TRTSLKTMSRRKLSQ |  | 87 | 429-443 | FNIEFRKAFLKILHC |
| **9** | 72 | 356-370 | KTMSRRKLSQQKEKK |  |  |  |  |
|  | 73 | 361-375 | RKLSQQKEKKATQML |  |  |  |  |
|  | 74 | 366-380 | QKEKKATQMLAIVLG |  |  |  |  |

AA = amino acid

**Supplementary table 2.** Deconvolution of D2R master pools previously identified to encompass the three T cell immunodominant regions. Each sub-pool comprises of two to three peptides.

| **Master pool** | **Sub-pool** | **Peptide** | | |
| --- | --- | --- | --- | --- |
| Pool 4 | A | 25 | 26 | 27 |
|  | B | 28 | 29 |  |
| Pool 5 | A | 32 | 33 | 34 |
|  | B | 35 | 36 | 37 |
| Pool 10 | A | 77 | 78 | 79 |
|  | B | 80 | 82 | 84 |
|  | C | 85 | 86 | 87 |

**Supplementary table 3.** HLA alleles of D2R-specific T cell-positive patients.

|  | **DRB1** | **DQA1** | **DQB1** | **DPA1** | **DPB1** |
| --- | --- | --- | --- | --- | --- |
| **Patient 1** | 04:03:01 | 01:03:01 | 03:02:01 | 02:02:02 | 05:01:01 |
|  | 08:03:02 | 03:01:01 | 06:01:01 | 02:^†^ | 135:01^‡^ |
| **Patient 2** | 12:01:01 | 01:02:01 | 03:01:01 | **01:03:01** | **04:01:01**^§^ |
|  | 13:02:01 | 05:05:01 | 06:09:01 | 01:03:01 | 104:01:01 |
| **Patient 3** | 11:01:01 | 01:03:01 | 3:01:01 | **01:03:01** | **04:01:01** |
|  | 13:01:01 | 05:05:01 | 06:03:01 | 01:03:01 | 04:01:01 |
| **Patient 4** | 03:01:01 | 01:02:01 | 02:01:01 | **01:03:01** | 01:01:01 |
|  | 15:01:01 | 05:01:01 | 06:02:01 | 02:01:02 | **04:01:01** |
| **Patient 5** | 01:02:01 | 01:01:02 | 02:02:01 | **01:03:01** | **04:01:01**^§^ |
|  | 07:01:01 | 02:01:01 | 05:01:01 | 02:01:01 | 11:01:01 |
| **Patient 6** | 04:05:01 | 03:02:01 | 03:03:02 | 2:02:02 | 02:02:01^§^ |
|  | 09:01:02 | 03:03:01 | 04:01:01 | 02:^†^ | 5:01:01 |
| **Patient 7** | 01:01:01 | 1:01:01 | 03:01:01 | **01:03:01** | **04:01:01** |
|  | 04:01:01 | 03:^†^ | 05:01:01 | 01:03:01 | 04:01:01 |
| **Patient 8** | 7:01:01 | 01:02:01 | 02:02:01 | **01:03:01** | **04:01:01** |
|  | 15:01:01 | 02:01:01 | 06:02:01 | 02:01:01 | 17:01:01 |

^†^ Novel alleles not yet documented in IEDB

^‡^ Allele not documented in IEDB

^§^ A rare combination of alleles is possible but this common and well documented combination of alleles is more likely and so considered for further analysis

In bold are haplotypes with high occurrence and co-exist in the same patient

**Supplementary table 4.** HLA alleles that occurred in greater than 25% of D2R-specific T cell-positive patients.

| **HLA allele** | **Occurrence in patients**  **(n=8)** |
| --- | --- |
| HLA-DPA1*01:03:01 | 6/8 (75%) |
| HLA-DPB1*04:01:01 | 6/8 (75%) |
| HLA-DQA1*01:02:01 | 3/8 (37.5%) |
| HLA-DQB1*03:01:01 | 3/8 (37.5%) |

**Supplementary table 5.** Antibody panel used for whole blood and PBMC CD25/OX40 assay.

| **Antigen** | **Fluorochrome** | **Clone** | **Company** |
| --- | --- | --- | --- |
| CD3 | V450 | UCHT1 | BD Biosciences |
| CD4 | FITC | RPA-T4 | BD Biosciences |
| CD25 | APC | 2A3 | BD Biosciences |
| CD134 | PE | L106 | BD Biosciences |

## Supplementary Figures

**Supplementary figure 1. T cell recognition of D2R peptides in patients with movement and psychiatric disorders was restricted to three master pools.** Activated D2R-specific T cells in whole blood from patients with movement and psychiatric disorders (n=24) of autoimmune (AI) and neurodevelopmental (ND) aetiology were detected using the CD25/OX40 assay. Samples were tested once soon after their collection to preserve sample integrity. When stimulated with the ten master pools of D2R peptides, pool 4, 5, and 10 elicited CD4^+^ T cell activation (Figure 1A). On the other hand, the frequency of activation by the remainder seven master pools in patients was comparable to controls (n=16). Dashed line indicates threshold defined as the mean+3SD of the controls.

**

**Supplementary figure 2. D2R-specific T cell activation is independent of D2R antibodies.** Patient sera (n=23) was assessed for D2R antibodies using the live cell-based assay with flow cytometry. 4/23 patients were seropositive for D2R antibodies as they exceeded the threshold (dotted line) defined as the mean+3SD. 0/8 D2R-specific T cell-positive patients were seropositive for D2R antibodies. Representative of three experiments shown.

**
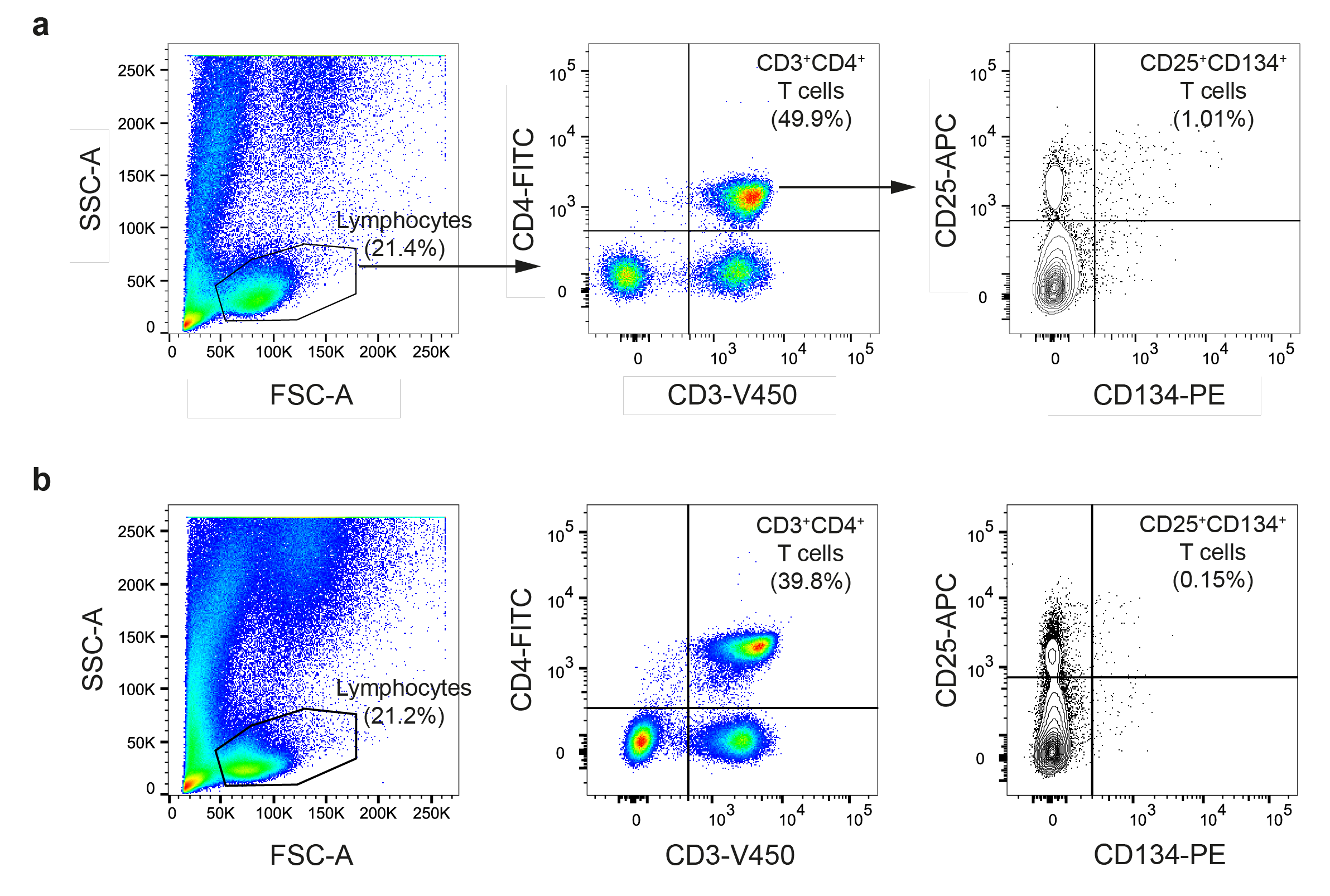
**

**Supplementary figure 3.** **(a)** Gating strategy used in the CD25/OX40 assay to identify activated antigen-specific T cells. Representative data shown of whole blood stimulated with tetanus toxoid. **(b)** Representative data of activated D2R-specific T cells in whole blood of Patient 8.
